# Dynamic Secondary Mitral Regurgitation: Current Evidence and Challenges for the Future

**DOI:** 10.3389/fcvm.2022.883450

**Published:** 2022-04-25

**Authors:** Hirokazu Onishi, Masaki Izumo, Toru Naganuma, Sunao Nakamura, Yoshihiro J. Akashi

**Affiliations:** ^1^Division of Cardiology, Department of Internal Medicine, St. Marianna University School of Medicine, Kawasaki, Japan; ^2^Department of Cardiology, New Tokyo Hospital, Matsudo, Japan

**Keywords:** dynamic secondary mitral regurgitation, heart failure with reduced left ventricular ejection fraction, guideline-directed medical therapy, cardiac resynchronization therapy, transcatheter edge-to-edge repair

## Abstract

Heart failure (HF) is a challenging situation in healthcare worldwide. Secondary mitral regurgitation (SMR) is a common condition in HF patients with reduced ejection fraction (HFrEF) and tends to be increasingly associated with unfavorable clinical outcomes as the severity of SMR increases. It is worth noting that SMR can deteriorate dynamically under stress. Over the past three decades, the characteristics of dynamic SMR have been studied. Dynamic SMR contributes to the reduction in exercise capacity and adverse clinical outcomes. Current guidelines refer to the indication of transcatheter edge-to-edge repair (TEER) for significant SMR based on data from the Cardiovascular Outcomes Assessment of the MitraClip Percutaneous Therapy for Heart Failure Patients with Functional Mitral Regurgitation (COAPT) trial if symptomatic despite optimal guideline-directed medical therapy (GDMT) and cardiac resynchronization therapy (CRT), but nonpharmacological treatment for dynamic SMR remains challenging. In HFrEF patients with LV dyssynchrony and dynamic SMR, CRT can improve LV dyssynchrony and subsequently attenuate SMR at rest and during exercise. Also, a recent study suggests that TEER with GDMT and CRT is more effective in symptomatic patients with HFrEF and dynamic SMR than GDMT and CRT alone. Further studies are needed to evaluate the safety and efficacy of nonpharmacological treatments for dynamic SMR. In this review, current evidence and challenges for the future of dynamic SMR are discussed.

## Introduction

Heart failure (HF) is a challenging situation in healthcare worldwide (1–3). HF with reduced ejection fraction (HFrEF) is seen in approximately half of the patients with HF (4). Secondary mitral regurgitation (SMR) with structurally normal leaflets is a common disease in patients with HFrEF (5, 6). Moreover, as the severity of SMR increases, the condition significantly tends to be incrementally associated with unfavorable clinical outcomes (6). As for the treatments for HFrEF with severe SMR, maximally tolerated guideline-directed medical therapy (GDMT) s is recommended (7–10). In patients with an ischemic etiology of the condition, the revascularization of significant coronary artery disease is recommended if applicable. Also, cardiac resynchronization therapy (CRT) for left ventricular (LV) dyssynchrony should be considered when the condition is refractory to the treatments above. Moreover, current guidelines recommend transcatheter edge-to-edge repair (TEER) if feasible when patients with HFrEF and severe SMR have symptoms despite optimal GDMT and CRT.

**Figure 1:**
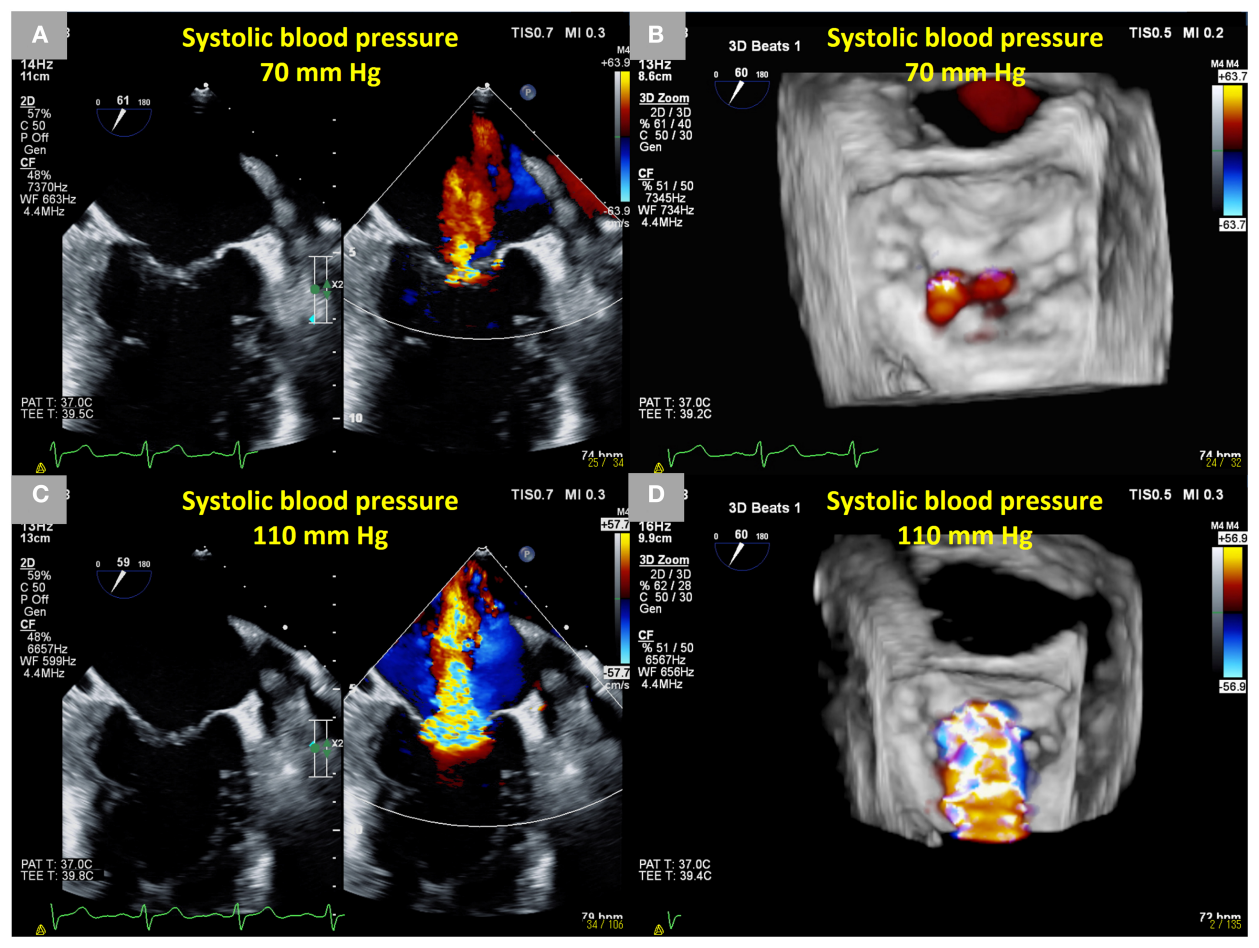
Dynamic changes in SMR during transesophageal echocardiography in a 66-year-old male patient who had an anterior old myocardial infarction and heart failure with reduced ejection fraction. Mild SMR (EROA 0.10 cm^2^) under sedation using midazolam under a systolic blood pressure of approximately 70 mm Hg. **(A)** Two-dimensional B-mode and color Doppler images from the intercommissural view and **(B)** a three-dimensional color Doppler image from the en-face view. Dynamic severe SMR (EROA 0.51 cm^2^) under an elevated systolic blood pressure of approximately 110 mm Hg using norepinephrine. **(C)** Two-dimensional B-mode and color Doppler images from the intercommissural view and **(D)** a three-dimensional color Doppler image from the en-face view. SMR, secondary mitral regurgitation; EROA, effective regurgitant orifice area.

It is worth noting that SMR can deteriorate dynamically according to changes in hemodynamics (Figure 1). The characteristics of dynamic SMR have been investigated during the past three decades (11–13). Dynamic SMR contributes to reductions in exercise capacity and adverse clinical outcomes (14–19). Although there are evidence-based nonpharmacological approaches for symptomatic SMR, the optimal treatment of dynamic SMR remains a matter of debate. In HFrEF patients with LV dyssynchrony and dynamic SMR, CRT can ameliorate LV dyssynchrony and subsequently attenuate dynamic SMR during exercise (20–22). Also, a recent study has demonstrated that TEER may be effective for dynamic severe SMR (23). Therefore, it is time to renew our knowledge of dynamic SMR and reconsider the optimal therapy of symptomatic dynamic SMR.

This review summarizes current evidence and challenges for the future of dynamic SMR in light of the mechanisms of dynamic SMR, its prognostic value, and its potentially effective treatment options.

## Mechanism of Dynamic SMR

The mitral valve apparatus is intricately comprised of several components, including the mitral annulus, anterior and posterior mitral leaflets, chordae tendinae, anterolateral and posteromedial papillary muscles, and adjacent LV wall. MR can be regulated based on an exquisite balance among these components during systole. In SMR, tethering and closing forces of the mitral valve during systole are essential to understand the intricate mechanism. The tethering force is affected by LV dilatation, LV sphericity, LV regional wall motion abnormalities, papillary muscle displacement, papillary muscle dyssynchrony, papillary muscle asymmetry, annular dilatation, and annular flattening. The closing force is decreased due to LV contractility impairments, LV dyssynchrony, increased left atrial (LA) pressure, and decreased mitral annular contraction.

Over the past three decades, the characteristics of dynamic SMR have been studied (11–13). Previous reports have elucidated that changes in LV dyssynchrony, LV sphericity, LV regional wall motion abnormality, increased mitral valve coaptation depth and tenting area, and mitral annular dilatation during exercise are crucial in light of the mechanism of dynamic SMR (24–32). It is worth noting that when comparing ischemic cardiomyopathy with apical and inferobasal scars, the coaptation depth is important in the case of an anterior myocardial infarction while the tenting area and LV regional wall motion abnormality are crucial in the case of an inferior myocardial infarction (24). There is a paucity of data on resting factors associated with dynamic SMR although only LV dyssynchrony at rest is suggested to be related to dynamic SMR (31). It may be because mitral valve tethering and closing forces change based on complicated combinations of dynamic changes of LV and LA geometry and MV apparatus during exercise.

## Exercise Capacity and Clinical Outcomes in Dynamic SMR

**Figure 2:**
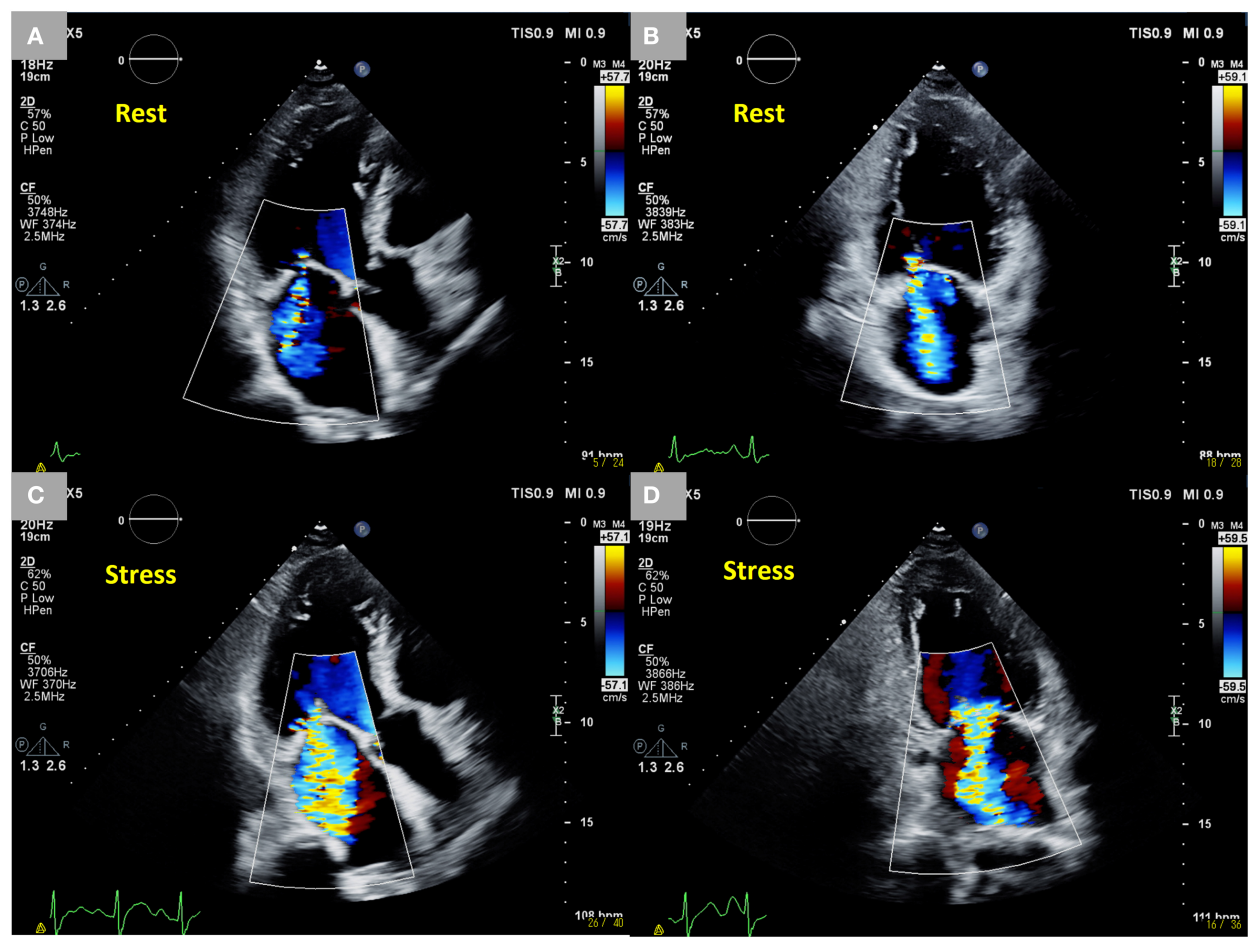
Dynamic changes in SMR during exercise stress echocardiography in an 85-year-old male patient who had an anterior old myocardial infarction and heart failure with reduced ejection fraction. Moderate SMR (EROA 0.22 cm^2^) at rest. **(A,B)** Two-dimensional color Doppler images from three- and two-chamber views. Dynamic severe SMR (EROA 0.46 cm^2^) under stress. **(C,D)** Two-dimensional color Doppler images from three-chamber and two-chamber views. SMR, secondary mitral regurgitation; EROA, effective regurgitant orifice area.

SMR can deteriorate dynamically during exercise (Figure 2). Dynamic SMR is expected to affect a patient's exercise tolerance due to the abruptly deteriorated severity, leading to a reduction in the forward stroke volume and an increase in the overload on the LA and pulmonary circulation during exercise. Izumo et al. elucidated that changes in the effective regurgitant orifice area (EROA) of SMR during exercise stress echocardiography (ESE) are significantly associated with the peak VO_2_ and VE/VCO_2_ slope and that the rate of exercise termination is higher in patients with dynamic SMR (ΔEROA ≥0.13 cm^2^ during exercise) than in those without dynamic SMR (18). Also, Bandera et al. investigated the exercise capacity of patients with HFrEF via cardiopulmonary exercise testing combined with ESE and reported that the exercise tolerance of patients with dynamic severe SMR (EROA ≥0.20 cm^2^ during exercise) was less than that of patients without dynamic severe SMR (19).

Lancellotti et al. initially reported that dynamic SMR with changes in the EROA ≥0.13 cm^2^ during exercise was independently associated with adverse clinical outcomes at mid-term (14, 15). Moreover, long-term clinical outcomes in patients with dynamic severe SMR (EROA ≥0.20 cm^2^ during exercise) were shown to be unfavorable by Suzuki et al. (16). Also, Piérard et al. (33) investigated the association of dynamic SMR with acute pulmonary edema in patients who recently suffered from acute pulmonary edema and underwent ESE after the improvement of pulmonary edema. Then, changes in the EROA on exercise were reported to be significantly associated with recent acute pulmonary edema. Furthermore, in patients that require hospitalization for acute decompensated HF, dynamic severe SMR on hospitalization is expected to be similar to persistent severe SMR in light of favorable outcomes (17).

## Pharmacological Treatment for Dynamic SMR

The four classes of drugs that constitute GDMT in HFrEF are angiotensin-converting enzyme inhibitors (ACEi)/angiotensin II receptor blockers (ARB)/angiotensin receptor-neprilysin inhibitors (ARNI), beta-blockers (BB), mineralocorticoid receptor agonists (MRA), and sodium-glucose cotransporter 2 inhibitors (SGLT2i); these drugs should be titrated to the maximum tolerated doses in all patients with HFrEF regardless of the presence of SMR (7–10). The optimization of GDMT using ACEi/ARB, BB, and MRA in HFrEF patients is expected to reduce the severity of SMR (34, 35). Also, ARNI has recently received attention as an effective basic HF drug and is recommended prior to ACEi/ARB in patients with HFrEF if applicable according to current guidelines (7–10). ARNIs are effective for LV and LA reverse remodeling (36–39). Moreover, ARNIs are reported to reduce the SMR (40). Also, according to data from a previous meta-analysis, the new “golden triangle” consisting of ARNIs, BBs, and MRAs is the most effective remedy for LV reverse remodeling among several combinations using some GDMT drugs (ACEi, ARB, ARNI, BB, and MRA) (41), which may be expected to bring about further improvements in the dynamic SMR.

A meta-analysis of three cardiac magnetic resonance imaging trials reported that SGLT2i therapy in patients with HFrEF was not significantly associated with reverse cardiac remodeling, including left ventricular ejection fraction, end-systolic volume, and end-diastolic volume; however, there was a tendency toward the improvement of these parameters (42).

## Nonpharmacological Treatment for Dynamic SMR

In HFrEF patients with persistent severe SMR, GDMT should be optimized as much as possible; however, such patients often suffer from either residual or worsening HF symptoms or undergo repeat HF hospitalization. Thus, when these patients have symptoms despite optimal GDMT, current guidelines recommend nonpharmacological treatment, including CRT and TEER (if applicable), after appropriate revascularization for significant coronary artery disease. However, there remains a matter for consideration in terms of nonpharmacological treatment for dynamic SMR in patients with HFrEF. Then, the nonpharmacological treatment of dynamic SMR is discussed below with a focus on CRT and TEER.

## CRT For Dynamic SMR

**Figure 3:**
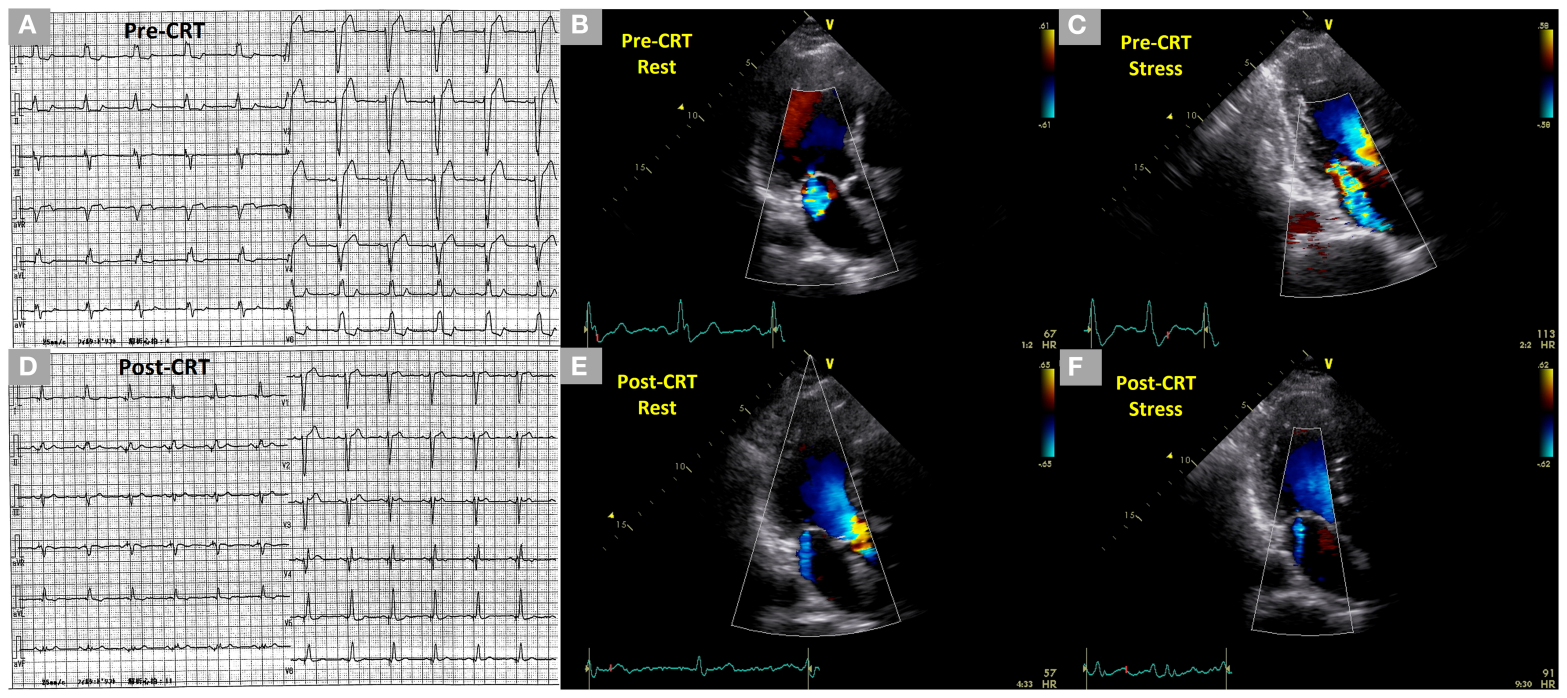
Dynamic changes of SMR during exercise stress echocardiography before CRT and controlled SMR following CRT during exercise stress echocardiography in a 71-year-old male patient who had an anterior old myocardial infarction and heart failure with reduced ejection fraction. **(A)** Complete left bundle branch block with a QRS duration of 154 ms in the electrocardiogram before CRT. **(B,C)** Mild SMR (EROA 0.14 cm^2^) at rest and dynamic moderate-to-severe SMR (EROA 0.32 cm^2^) under stress in two-dimensional color Doppler images from the three-chamber view before CRT. **(D)** Non-left bundle branch block with a QRS duration of 128 ms in the electrocardiogram after CRT. **(E,F)** Trivial SMR at rest and under stress 1 year after the CRT in two-dimensional color Doppler images from the three-chamber view after CRT. SMR, secondary mitral regurgitation; CRT, cardiac resynchronization therapy, EROA, effective regurgitant orifice area.

In HFrEF patients with LV dyssynchrony, CRT can suppress LV dyssynchrony and subsequently improve LV hemodynamics while attenuating MR at rest and during exercise (Figure 3) (20, 22). Madaric et al. elucidated the time course of changes in LV dyssynchrony, LV contractility, and SMR at rest and during exercise following CRT (21). Approximately 1 week after CRT, LV dyssynchrony and SMR during exercise did not adequately improve despite ameliorations in LV dyssynchrony and SMR at rest. However, approximately 3 months after CRT, LV dyssynchrony and dynamic SMR were controlled even during exercise with resting SMR and in LV volumes progressively reduced despite there being no additional improvement in the resting LV dyssynchrony. Moreover, the cardiopulmonary performance after CRT improved at late follow-up in HFrEF patients with dynamic SMR although no reports showed a prognostic value of CRT.

Also, it was (reportedly) possible to induce a left bundle branch block (LBBB) during exercise by increasing the heart rate of HFrEF patients with non-LBBB at rest (43). Rate-related LBBB may be accompanied by LV dyssynchrony and dynamic SMR, subsequently leading to deteriorated hemodynamics and worsening HF symptoms. In the case report, the patient underwent CRT to correct rate-related LBBB, LV dyssynchrony, and dynamic SMR and had favorable clinical outcomes during follow-up (43). Thus, dynamic SMR with rate-related LBBB may be assessed using ESE in unexplained symptomatic patients with HFrEF despite resting non-LBBB, no dyssynchrony, and non-significant SMR.

## TEER for Dynamic SMR

**Figure 4:**
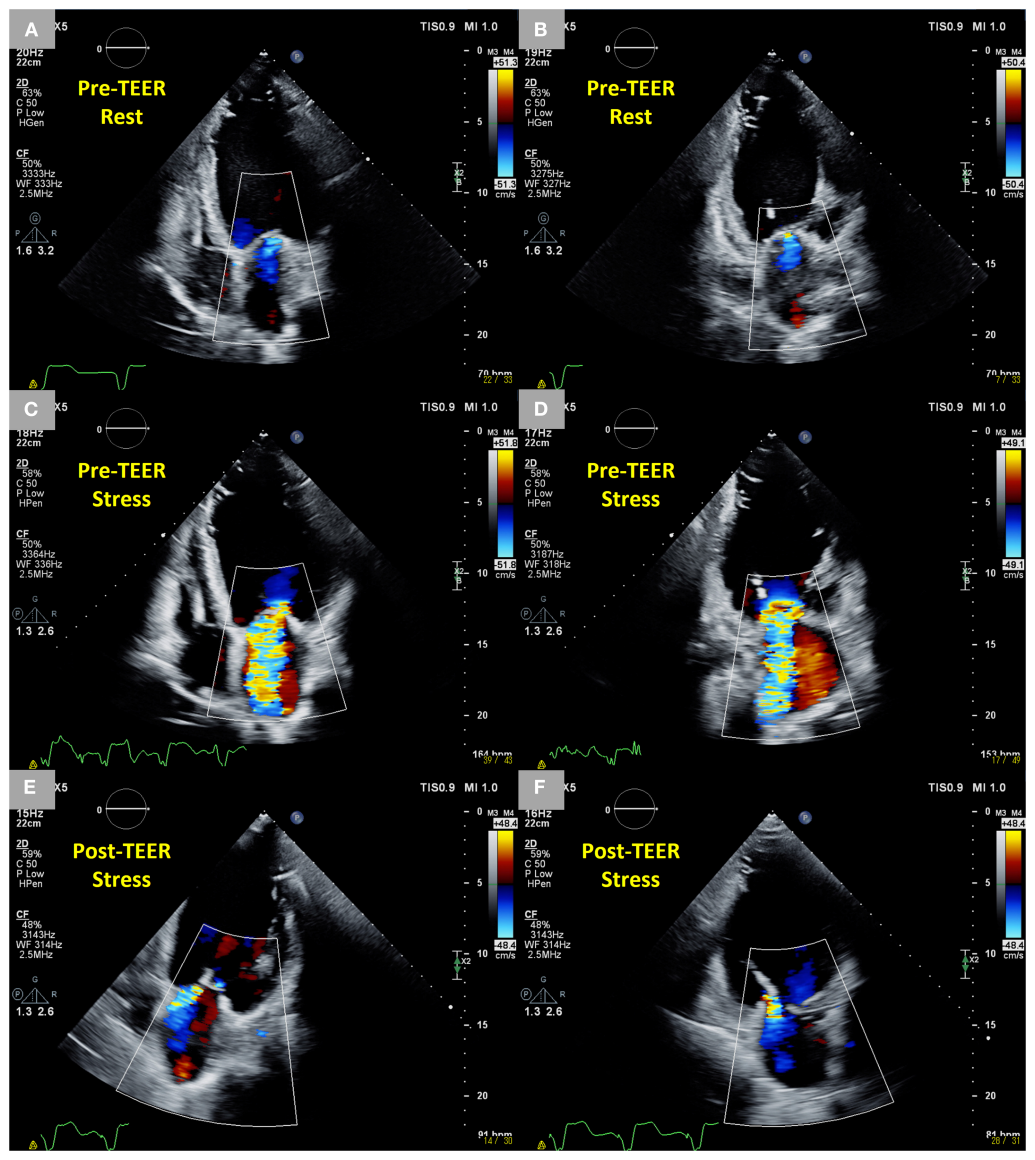
Dynamic changes in SMR during exercise stress echocardiography before TEER and controlled SMR following TEER during exercise stress echocardiography in an 83-year-old male patient who had an anterior and inferior old myocardial infarction and heart failure with reduced ejection fraction after CRT. **(A,B)** Trivial SMR at rest in two-dimensional color Doppler images from four-chamber and two-chamber views. **(C,D)** Dynamic severe SMR (EROA 0.52 cm^2^) under stress in two-dimensional color Doppler images from four-chamber and two-chamber views. **(E,F)** Mild MR under stress 6 months after the TEER in two-dimensional color Doppler images from four-chamber and two-chamber views. SMR, secondary mitral regurgitation; TEER, transcatheter edge-to-edge repair; CRT, cardiac resynchronization therapy; EROA, effective regurgitant orifice area; MR, mitral regurgitation.

In patients with HFrEF and dynamic SMR, it may be possible that HF symptoms remain or recur with dynamic SMR refractory to optimal GDMT and CRT. In such patients, TEER can control not only the SMR at rest but also the dynamic change during exercise (Figure 4). Previously, Lancellotti et al. suggested that dynamic SMR should be considered in HFrEF patients with moderate SMR at rest and unexplained dyspnea under optimal GDMT and nonpharmacological treatments, including CRT and revascularization, if indicated, and TEER might be indicated when dynamic SMR was performed during exercise (44). Recently, Izumo et al. reported that TEER is suggested to be safe and effective in light of HF symptoms and clinical outcomes during follow-up in symptomatic HFrEF patients with dynamic SMR (ΔEROA ≥0.13 cm^2^ during exercise) refractory to treatment with optimized GDMT and CRT if applicable (23). Of note, the patients in the non-TEER group, who had no significant SMR (the EROA of 0.20 ± 0.08 cm^2^) at rest but significant SMR (the EROA of 0.38 ± 0.10 cm^2^) during exercise, had a significantly higher rate of HF-related hospitalization and all-cause mortality than those in the TEER group. This suggested that resting SMR in patients with HFrEF is potentially underestimated unless ESE is performed and, consequently, even a non-significant resting SMR can bring about adverse clinical events if medically treated. In addition, this study addressed the association of EROA of SMR with left ventricular end-diastolic volume at rest and during exercise. In the study patients, EROA and left ventricular end-diastolic volume increased significantly during exercise, and as a result, the relationship between EROA in the study and left ventricular end-diastolic volume during exercise was similar to that in the COAPT study. Thus, ESE may be useful to figure out symptomatic patients with HFrEF who have potentially disproportionate dynamic SMR and are expected to receive adequate benefit from TEER.

TEER can reduce the intensity of the symptoms of SMR and its prevalence because of the acute changes in mitral valve geometry as follows; improved coaptation area and mitral valve tethering, decreased anteroposterior diameter and area of the mitral annulus, and increased sphericity of the mitral annulus (45–50), all of which lead to a persistent reduction of the SMR and improvement of the functional status (45, 46). Such acute changes following TEER seem to resist dynamic SMR derived from changes in the mitral valve geometry during exercise. Therefore, TEER could be a reasonable treatment for dynamic SMR in patients with HFrEF. Further studies are required to evaluate the safety and efficacy of TEER for dynamic SMR.

## Challenges for the Future in Dynamic SMR

Optimal GDMT reduces the severity of SMR in patients with HFrEF (34, 35, 40). Current guidelines recommend TEER for SMR in patients with HFrEF if they had HF symptoms despite the uptitration of HF drugs as long as tolerated (7, 8). However, a previous study reported that optimal GDMT before TEER was not necessarily achieved (51). Less than 50% of the overall population received >50% of the target dose of ACEi/ARB/ARNI and BB, which suggests the difficulty in the maximal optimization of GDMT in clinical practice. This might be because of hypotension, worsening HF, drug intolerance, and worsening kidney function.

The study also reported that 67% of the patients who underwent TEER had either unchanged or uptitrated GDMT (51). Such patients showed a lower rate of recurrent MR ≥3+, more reduced LV end-systolic volumes, and lower NYHA classes during follow-up than those with downtitrated GDMT. Moreover, unchanged or uptitrated GDMT following TEER was associated with favorable clinical outcomes, which was defined as freedom from death and heart transplantation.

TEER for dynamic SMR may be expected to improve hemodynamics, mitral valve geometry, and HF symptoms, subsequently enabling patients to avoid downtitrated GDMT and gain clinical benefits as with TEER for persistent severe SMR. Thus, optimal GDMT even after TEER for dynamic SMR is also considered the crucial cornerstone of HF management considering its effect in further cardiac reverse remodeling and SMR reduction (45–53). Further studies are needed in light of the importance of optimal GDMT after TEER as well as the safety and efficacy of TEER in patients with HFrEF and dynamic SMR.

Also, there are issues with ESE in patients with HFrEF and SMR; such patients can not exercise long enough to reach peak stress. Therefore, it may be difficult to compare dynamic SMRs among different patients based on certain stress criteria. Then, low-load ESE, which can be performed for a shorter time and under lower stress than conventional ESE, may be reasonable to evaluate the dynamic changes of SMR under specific stress in HFrEF patients. In such patients, it is expected that the usefulness of low load ESE will be examined in future.

## Conclusion

Dynamic SMR is associated with exercise performance impairments and adverse clinical outcomes in patients with HFrEF. In such patients, optimal GDMT and CRT are expected to ameliorate the deteriorated mitral valve apparatus, which subsequently leads to improvements in the dynamic SMR. Dynamic SMR can be residual or recurrent even after administering the appropriate treatments above. In such cases, other invasive treatment options, including TEER, may be indicated considering the effectiveness of TEER for dynamic SMR. CRT and TEER, along with GDMT, can improve deteriorated mitral valves and LVs, left ventricular dynamics, HF symptoms, exercise tolerance, and clinical outcomes. Even after obtaining such clinical benefits from CRT and TEER, GDMT regimens should be re-evaluated and reinforced as long as patients are tolerated to aim at further cardiac reverse remodeling and the reduction and prevention of dynamic SMR, subsequently leading to the amelioration of exercise tolerance and clinical outcomes.

## Author Contributions

HO and MI drafted the manuscript and prepared the figures. TN, SN, and YA revised the manuscript. All authors read and approved the final manuscript.

## Conflict of Interest

The authors declare that the research was conducted in the absence of any commercial or financial relationships that could be construed as a potential conflict of interest.

## Publisher's Note

All claims expressed in this article are solely those of the authors and do not necessarily represent those of their affiliated organizations, or those of the publisher, the editors and the reviewers. Any product that may be evaluated in this article, or claim that may be made by its manufacturer, is not guaranteed or endorsed by the publisher.
